# MFI2 upregulation promotes malignant progression through EGF/FAK signaling in oral cavity squamous cell carcinoma

**DOI:** 10.1186/s12935-023-02956-0

**Published:** 2023-06-12

**Authors:** Wei-Chen Yen, Kai-Ping Chang, Cheng-Yi Chen, Yenlin Huang, Ting-Wen Chen, Hsing-Wen Cheng, Jui-Shan Yi, Chun-Chia Cheng, Chih-Ching Wu, Chun-I Wang

**Affiliations:** 1 Department of Otolaryngology-Head & Neck Surgery, Chang Gung Memorial Hospital, Taoyuan, Taiwan; 2 Molecular Medicine Research Center, Chang Gung University, Taoyuan, Taiwan; 3 College of Medicine, Chang Gung University, Taoyuan, Taiwan; 4 Department of Cell Biology and Anatomy, College of Medicine, National Cheng Kung University, Tainan, Taiwan; 5 School of Medicine, National Tsing-Hua University, Hsinchu, Taiwan; 6 Institute of Stem Cell and Translational Cancer Research, Department of Anatomic Pathology, Chang Gung Memorial Hospital at Linkou, Taoyuan, Taiwan; 7 Institute of Bioinformatics and Systems Biology, National Yang Ming Chiao Tung University, Hsinchu, Taiwan; 8 Department of Biological Science and Technology, National Yang Ming Chiao Tung University, Hsinchu, Taiwan; 9 Center For Intelligent Drug Systems and Smart Bio-devices (IDS2B), National Yang Ming Chiao Tung University, Hsinchu, Taiwan; 10 Radiation Biology Research Center, Institute for Radiological Research, Chang Gung University, Taoyuan, Taiwan; 11 Department of Medical Biotechnology and Laboratory Sciences, College of Medicine, Chang Gung University, Taoyuan, Taiwan; 12 Department of Biochemistry, School of Medicine, China Medical University, Taichung, Taiwan

## Abstract

Oral squamous cell carcinoma (OSCC) is the predominant histological type of the head and neck squamous cell carcinoma (HNSCC). By comparing the differentially expressed genes (DEGs) in OSCC-TCGA patients with copy number variations (CNVs) that we identify in OSCC-OncoScan dataset, we herein identified 37 dysregulated candidate genes. Among these potential candidate genes, 26 have been previously reported as dysregulated proteins or genes in HNSCC. Among 11 novel candidates, the overall survival analysis revealed that melanotransferrin (MFI2) is the most significant prognostic molecular in OSCC-TCGA patients. Another independent Taiwanese cohort confirmed that higher MFI2 transcript levels were significantly associated with poor prognosis. Mechanistically, we found that knockdown of MFI2 reduced cell viability, migration and invasion via modulating EGF/FAK signaling in OSCC cells. Collectively, our results support a mechanistic understanding of a novel role for MFI2 in promoting cell invasiveness in OSCC.

**Supplementary Information:**

The online version contains supplementary material available at 10.1186/s12935-023-02956-0.

## Introduction

Oral squamous cell carcinoma (OSCC) is one of the most common malignant diseases of the head and neck, accounting for about 370,000 new incidence and about 170,000 mortality in 2020 worldwide. Although there has been significant progress in all major therapeutic approaches [1, 2], the 5-year overall survival rate of OSCC has not obviously increased [3, 4]. OSCC carcinogenesis stems from exposure to environmental carcinogens, including cigarette smoking, alcohol consumption and betel quid chewing, genomic aberrations and widespread genomic instability [5, 6]. To improve the detection and treatment of OSCC, the identification of novel and effective prognostic and predictive factors may increase our knowledge of OSCC tumorigenesis and uncover the underlying mechanisms.

To identify a potential biomarker for OSCC, we identified unique copy number variations (CNVs) in tumors of Taiwanese patients with OSCC [7]. In the present study, we performed bioinformatic analyses based on high‐throughput RNA sequencing of OSCC from The Cancer Genome Atlas (TCGA) (http://cancergenom-e.nih.gov/) to identify differentially expressed genes (DEGs) between normal and OSCC patients. We compared the DEGs with CNVs that we identified previously and recognized a novel oncogene, Melanotransferrin (MFI2), involved in OSCC carcinogenesis.

MFI2, also known as MTF, CD228 and melanoma-associated antigen p97, is a homolog of the serum iron transport protein transferrin (TF) [8, 9]. The protein name is derived from its sequence, which is similar to the TF superfamily, and its ability to bind iron. The similarities between MFI2 and TF, together with their high expression in melanoma cells, led to the hypothesis that MFI2 may play a role in iron uptake by tumor cells [10]. However, studies have shown that the MFI2 protein contains only one iron-binding site at the N-terminus and has been demonstrated to play little role in iron uptake by melanoma cells [11]. Various studies suggested roles of MFI2 in endometrial regeneration [12], melanoma cell proliferation and migration [13, 14], plasminogen activation [15, 16], differentiation [17] and the transport of iron across the blood–brain barrier [18].

The expression of MFI2 in normal tissues is lower than that in tumor tissues and embryo tissues. Previous studies indicated that MFI2 is mainly expressed in melanoma and is associated with tumor metastasis and angiogenesis. Aberrant upregulation of MFI2 has also been observed in colorectal cancer and gastric cancer and is associated with a poor prognosis [19–22]. However, the understanding of the roles of dysregulated MFI2 in OSCC cells remains very limited. The current study aimed to investigate the clinicopathological associations and underlying mechanisms of MFI2-mediated cell invasiveness in OSCC cells.

## Materials and methods

### Patient populations and clinical specimens

Tumor specimens and pericancerous normal tissues for real-time quantitative PCR (qPCR) analysis were obtained from a testing cohort, including 115 patients who were surgically resected and enrolled consecutively among the diagnosed OSCC patients from 2006 to 2013. The patients in this study underwent standard preoperative assessments and follow-up according to the institutional guidelines as described previously [7]. This study was approved by the Institutional Review Board at Chang Gung Memorial Hospital, Taiwan (IRB no. 202001603B0).

### Identification of differentially expressed genes (DEGs) in OSCC from the TCGA dataset

The expression levels of mRNA in the TCGA-OSCC dataset were downloaded from Broad GDAC Firehose (https://gdac.broadinstitute.org), including 315 OSCC tumors and 30 normal samples. Transcripts per kilobase million values representing mRNA expression calculated from RNA-Seq data by expectation maximization (RSEM) [23] were used for DEG detection with Partek Genomics Suite software (Inc. P. Partek Genomics Suite, St. Louis). Through this analysis, we identified 4789 genes that were significantly differentially expressed in tumors compared to normal tissues (twofold change and* p* < 0.05). ANOVA were applied to detect differentially expressed genes from log transformed expressed level.

### Cell culture

KOSC3 cells were cultured in RPMI 1640 (Invitrogen, Carlsbad, CA) supplemented with 10% fetal bovine serum (FBS) (Gibco BRL, Carlsbad, MD, USA), 100 units/ml penicillin and 100 μg/ml streptomycin (Gibco). SAS cells were maintained in Dulbecco’s modified Eagle’s medium (DMEM) (Invitrogen) containing 10% FBS plus antibiotics; SCC4 cells were cultured in DMEM/F12 (Invitrogen) containing 10% FBS plus antibiotics and 400 ng/ml hydrocortisone. The cells were cultured at 37 °C in a humidified atmosphere of 95% air and 5% CO_2_.

### Small interfering RNA (siRNA) transfection

Briefly, siRNA targeting human MFI2 was purchased from Dharmacon (Thermo Fisher Scientific, Rockford, IL). OSCC cells were transfected with the Dharmacon ON-TARGETplus Nontargeting Control Pool (Thermo Fisher Scientific) or MFI2-pooled siRNA (GGUGAUGGGCUGCGAUGUA, GGGCGAAGUGUACGAUCAA, GGGCAGGAGAGACCAGUUA and GCACGGUACUGGAGAACAC) using RNAiMAX (Invitrogen) based on the manufacturer’s instructions.

### Transfection of plasmids

The cDNA encoding MFI2 (Accession No. NM_005929.6) was cloned into the pcDNA3.1+/C-(K)-DYK plasmid (GenScript, USA). OSCC cells were seeded in 6-well plates and transfected with MFI2 plasmids (0.75 μg) using Lipofectamine 2000 (Invitrogen) based on the manufacturer’s instructions.

### RNA extraction and quantitative reverse transcription polymerase chain reaction

Total RNA was extracted from OSCC tumor and normal counterpart tissues, and cDNA was prepared for qPCR using commercially available primers (MFI2 Hs00195551_m1 and normalization control ACTB, Hs01060665_g1; Assay-on-Demand, Applied Biosystems, Foster City, CA) as described previously [7].

### Cell migration and invasion assay

After transfection, the cells were harvested by trypsinization and suspended in serum-free culture medium. For the migration assay, the cells (300 μl; 1 × 10^4^ cells) were added to the upper chambers of 24-well Transwell plates (0.8 μm pore size filter; Corning, Canton, NY). For the invasion assay, the upper chambers of 24-well Transwell plates were coated with Matrigel™ Basement Membrane Matrix (BD Biosciences, San Jose, CA) at 37 °C for 2 h. The cells (200 μl; 1 × 10^4^ cells) were suspended in of serum-free culture medium and added to the upper chamber. After a 24 h incubation at 37 °C, the chambers were washed, fixed, stained and counted.

### Cell proliferation assay

After transfection for 24 h, OSCC cells were harvested by trypsinization and suspended at a density of 3 × 10^2^ (SCC4) and 5 × 10^2^ cells/100 μl (KOSC3) in a 96-well plate (100 μl per well). Cell viability was evaluated with Cell Counting Kit-8 (CCK-8) (BIOTOOLS Co., Ltd. Taiwan) according to the manufacturer’s protocol. Briefly, cells in each well were incubated with 10 µl CCK‑8 reagent at 37 °C for 2 h. The optical density was measured at a wavelength of 450 nm using an ELISA reader (Molecular Devices, SpectraMax M2).

### Western blot

The total protein in the lysates and supernatants was analyzed by western blotting. The cells were collected using lysis buffer, and the protein concentration was determined by the Bradford assay. The protein in the supernatants was concentrated by trichloroacetic acid (TCA) precipitation. Protein samples were denatured at 95 °C, resolved on SDS-polyacrylamide gels, and transferred onto PVDF membranes. The membranes were incubated overnight at 4 °C with an appropriate dilution of the indicated primary antibody. The membranes were then incubated with an appropriate dilution of an HRP-conjugated secondary antibody for 1 h. The immune reactive bands were exposed by the use of ECL reagents, and the signals were captured by X-ray films. The intensity of the bands was quantified by using ImageJ software. β-Actin was used as a loading control. The indicated antibodies against the following proteins were used for Western blotting: anti-FAK, anti-pFAK, anti-Src, anti-pSrc, anti-AKT and anti-pAKT were all purchased from Cell Signaling Technology (Beverly, MA, USA). Anti-EGF were purchased from R&D Systems (Minneapolis, MN). Anti-MFI2 were purchased from Novus Biologicals (USA).

### Statistical analysis

The Wilcoxon test was used to analyze the qPCR results from OSCC and normal counterpart tissues. The results of the migration and invasion assays and mRNA expression in the OSCC cell lines were analyzed using the nonparametric Mann–Whitney *U* test. Chi-square tests were used to determine the differences between MFI2 expression and various clinicopathologic factors. Two-tailed *p* values of 0.05 or less were considered significant. A comparison of survival rates was plotted using the Kaplan–Meier method and examined by the log-rank test. Statistical analyses were performed using GraphPad Prism V5.01 (GraphPad Software, Inc., San Diego, CA, USA).

## Results

### Elevated MFI2 levels in patients with OSCC are correlated with a poor prognosis

**Fig. 1:**
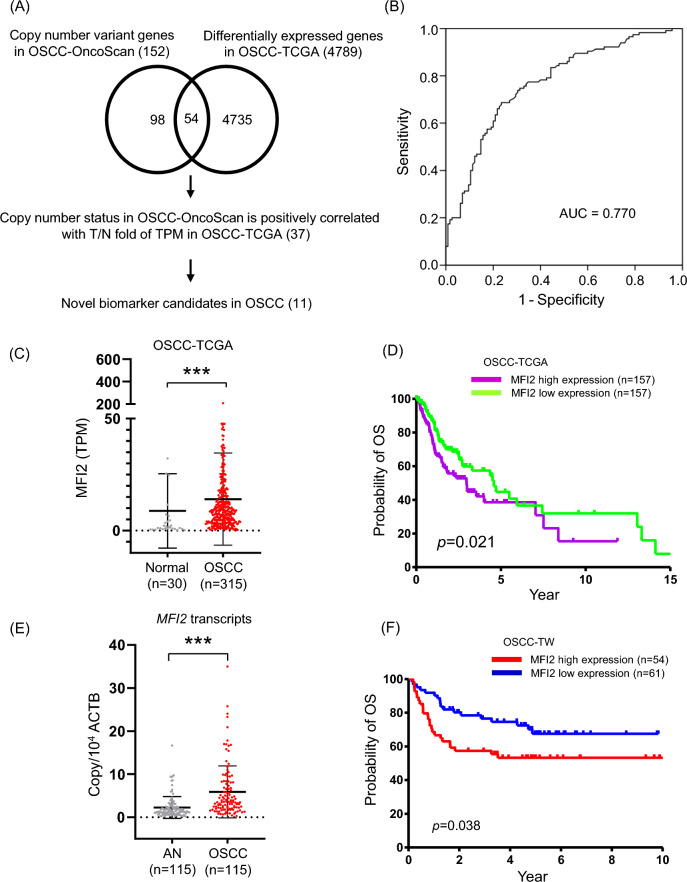
Association of high MFI2 expression with shorter patient survival in OSCC. **A** Workflow for the candidate selection of OSCC from the OncoScan and TCGA databases. **B** The ROC curve of MFI2. The value of AUC was 0.770. **C** Transcript expression levels of MFI2 in the OSCC-TCGA dataset. **D** Kaplan–Meier plot for overall survival (OS) stratified by the expression of MFI2 among the 314 patients in the OSCC-TCGA dataset (*p* = 0.021). **E** MFI2 transcripts in 115 paired OSCC tissues were determined by qPCR. **F** Kaplan–Meier plot showing the OS rates for patient subgroups stratified by high versus low MFI2 expression (*p* = 0.038). The *p* values were calculated by using log-rank tests

To identify novel diagnosis- or metastasis-related genes that are dysregulated in OSCC, we identified DEGs in OSCC from the TCGA dataset and compared the DEGs with the copy number variant genes that we identified in the OSCC-OncoScan dataset previously [7]. With this comparison, we found 54 genes were overlapped. Among this, 37 potential candidate genes that comprised a positive correlation between copy number status and T/N fold of transcripts per million. Among these potential candidate genes, 26 have been previously reported as dysregulated proteins or genes in head and neck squamous cell carcinoma (HNSCC) (Fig. 1A and Table 1). The receiver operating characteristic (ROC) was used to evaluate the utility of target as biomarker for OSCC by calculating the area under the ROC curve (AUC). The AUC value of MFI2 was 0.770, which indicated a high ability to differentiate OSCC from healthy control (Fig. 1B). To elucidate the clinical association with the 11 novel candidates, we determined the overall survival (OS) of the 11 candidates in OSCC-TCGA and found only the survival between high and low expressed MFI2 were significantly different in the OSCC-TCGA dataset. As shown in Fig. 1C, MFI2 expression was increased in OSCC tumors compared with normal tissues in OSCC-TCGA. The OS rate was significantly different between high and low expression of MFI2 in the OSCC-TCGA dataset (Fig. 1D).

**Table 1:** List of 37 differentially expressed genes in OSCC via comparison of the OSCC-OncoScan and OSCC-TCGA-DEG datasets

Gene name	CNV in OncoScan	T/N in OSCC-TCGA	Dysregulated in HNSCC (ref.)	*p* value^a^ of OS rate in OSCC-TCGA
CSMD1	Del	0.10	Yes [45]	
DOCK3	Del	0.32		0.14
FHIT	Del	0.14	Yes [46]	
KAT2B	Del	0.31		0.58
LPL	Del	0.30	Yes [47]	
MITF	Del	0.29	Yes [48]	
NAT2	Del	0.06	Yes [49]	
PPARG	Del	0.11		0.21
ROBO2	Del	0.06	Yes [50]	
SCARA5	Del	0.18		0.098
SLC7A2	Del	0.20		0.79
THRB	Del	0.44	Yes [51]	
ATP6V1C1	Amp	2.03	Yes [52]	
CSMD3	Amp	66.29	Yes [53]	
E2F1	Amp	3.57	Yes [54]	
EIF5A2	Amp	3.41	Yes [55]	
EXT1	Amp	3.20	Yes [56]	
FANCC	Amp	2.06	Yes [57]	
FANCG	Amp	2.14	Yes [58]	
GLI3	Amp	2.08		0.78
HECW1	Amp	2.36		0.48
HIF1A	Amp	2.02	Yes [59]	
IGFBP1	Amp	78.17	Yes [60]	
IGFBP3	Amp	3.03	Yes [61]	
INHBA	Amp	28.45	Yes [62]	
LEPREL1	Amp	4.54		0.32
MFI2	Amp	2.64		0.021^b^
PAX5	Amp	11.83	Yes [63]	
PCNA	Amp	2.06	Yes [64]	
PLEC	Amp	2.18	Yes [65]	
PRKDC	Amp	2.00	Yes [66]	
RCOR2	Amp	2.59		0.23
RECQL4	Amp	3.26	Yes [67]	
ROR2	Amp	3.46	Yes [68]	
SOX12	Amp	2.34		0.10
TFRC	Amp	2.27	Yes [69]	
TP63	Amp	2.53	Yes [70]	

Amp., amplification; CNV, copy number variation; Del., deletion; T/N, tumor tissue sample and normal tissue sample; TCGA, The Cancer Genome Atlas; HNSCC, Head and neck squamous cell carcinoma

^a^*p* value is determined by the log-rank tests

^b^These are considered statistically significant

Accordingly, overexpression of MFI2 in OSCC tumors was additionally confirmed in another independent Taiwanese cohort. We examined the mRNA expression of MFI2 in 115 OSCC tissue specimens containing tumors and their adjacent normal tissues. These results consistently showed that the mRNA level of MFI2 was increased in OSCC tumors compared with adjacent normal tissues (Fig. 1E). Clinicopathological analysis demonstrated that the MFI2 expression levels in OSCC tumors were positively associated with node classification, overall TNM stage and perineural invasion (*p* = 0.011, 0.015 and 0.012, respectively). In contrast, the MFI2 expression level and other parameters showed no significant association with sex, age, tumor classification, extranodal extension, or differentiation (Table 2). Consistently, patients with high MFI2 expression presented significantly shorter OS than those with low MFI2 expression, and the 5-year OS rates were 53.7% and 70.5% for patients with high and low MFI2 expression, respectively (Fig. 1F).

**Table 2:** The clinicopathological characteristics related to the expression of MFI2 in 115 samples of OSCC

Patient categories	Case number	MFI2 expression level		
		Low (%)	High (%)	*p* value
Sex				
Male	103	56 (54.4)	47 (45.6)	0.404
Female	12	5 (41.7)	7 (58.3)	
Age^a^		51.0 ± 10.8 (78.3, 22.5)	52.8 ± 13.4 (79.8, 20.3)	0.550
Tumor classification				
T1–T2	58	32 (55.2)	26 (44.8)	0.644
T3–T4	57	29 (50.9)	28 (49.1)	
Node classification				
0	55	36 (65.5)	19 (34.5)	0.011^b^
> 0	60	25 (41.7)	35 (58.3)	
Overall TNM stage				
I–II	34	24 (70.6)	10 (29.4)	0.015^b^
III–IV	81	37 (45.7)	44 (54.3)	
ENE				
No	84	48 (57.1)	36 (42.9)	0.147
Yes	31	13 (41.9)	18 (58.1)	
PNI				
No	59	38 (64.4)	21 (35.6)	0.012^b^
Yes	56	23 (41.1)	33 (58.9)	
Differentiation				
Well + moderately	100	56 (56.0)	44 (44.0)	0.101
Poorly	15	5 (33.3)	10 (66.7)	
Tumor depth (mm)				
≤ 8	44	24 (54.5)	20 (45.5)	0.799
> 8	71	37 (52.1)	34 (47.9)	

ENE, extranodal extension; PNI, perineural invasion

^a^Mean ± SD, (maximum, minimum)

^b^These are considered statistically significant

### MFI2 is involved in cell proliferation, migration and invasion in OSCC cells

**Fig. 2:**
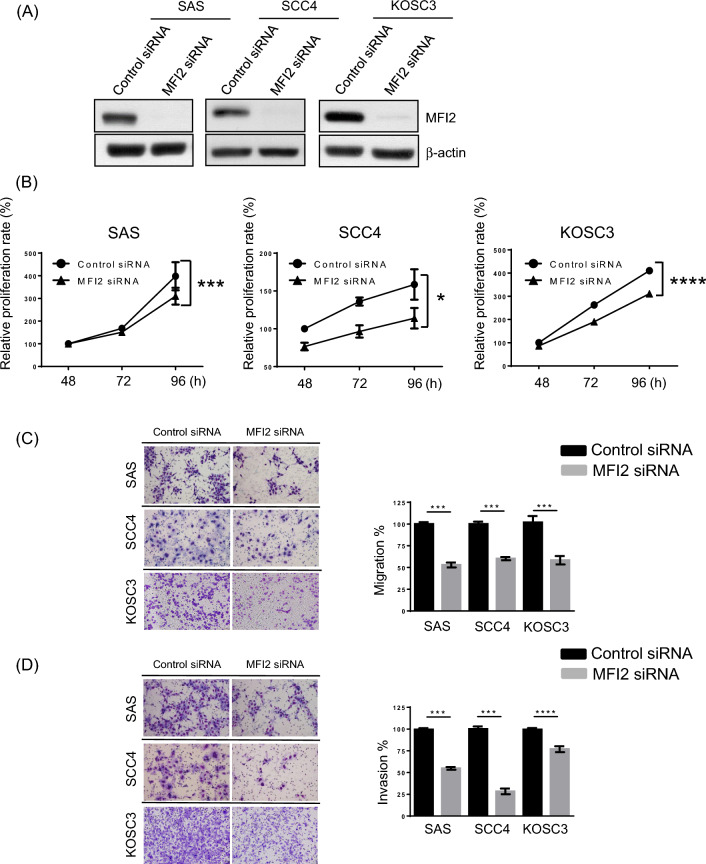
MFI2 is involved in the migration and invasive ability of OSCC cells. **A** SAS, SCC4 and KOSC3 cells were transfected with control siRNA and MFI2-specific siRNA. The protein expression of MFI2 was analyzed by western blot. β-Actin was used as the loading control. **B** Cell proliferation assay (CCK-8) demonstrated that MFI2-knockdown inhibited the proliferation of SAS, SCC4 and KOSC3 cells. Data are presented as the mean of three experiments. The transfected cells were subjected to migration (**C**) and invasion (**D**) assays. Representative microphotographs of the filters obtained from the migration and invasion assays. Original magnification: ×100 (left). Quantitative analysis of the migration and invasion assays (right). Data are presented as the mean values obtained from three independent experiments

Clinicopathological analysis clearly indicated that MFI2 is involved in tumor cell invasiveness. To examine the possible roles of MFI2 in the malignant progression of OSCC, we applied a siRNA approach to suppress the expression of endogenous MFI2 in SAS, SCC4 and KOSC3 cells and assessed the effects on cell proliferation, migration and invasion. Western blotting showed that MFI2 protein levels were significantly reduced in cells transfected with MFI2 siRNA compared with control siRNA (Fig. 2A). The CCK-8 assay revealed that cell proliferation was decreased in MFI2-knockdown SAS, SCC4 and KOSC3 cells (Fig. 2B). In addition, the Transwell migration assay showed that the migration ability of MFI2-knockdown SAS, SCC4 and KOSC3 cells was decreased compared with that of the control cells (Fig. 2C). The Transwell invasion assay further demonstrated that the invasion ability was significantly impaired in MFI2-knockdown SAS (*p* = 0.0005), SCC4 (*p* = 0.0418) and KOSC3 (*p* < 0.0001) cells (Fig. 2D). Collectively, these results indicate that MFI2 is involved in OSCC cell proliferation, migration and invasiveness.

### MFI2 is positively correlated with the level of EGF in OSCC cells

**Fig. 3:**
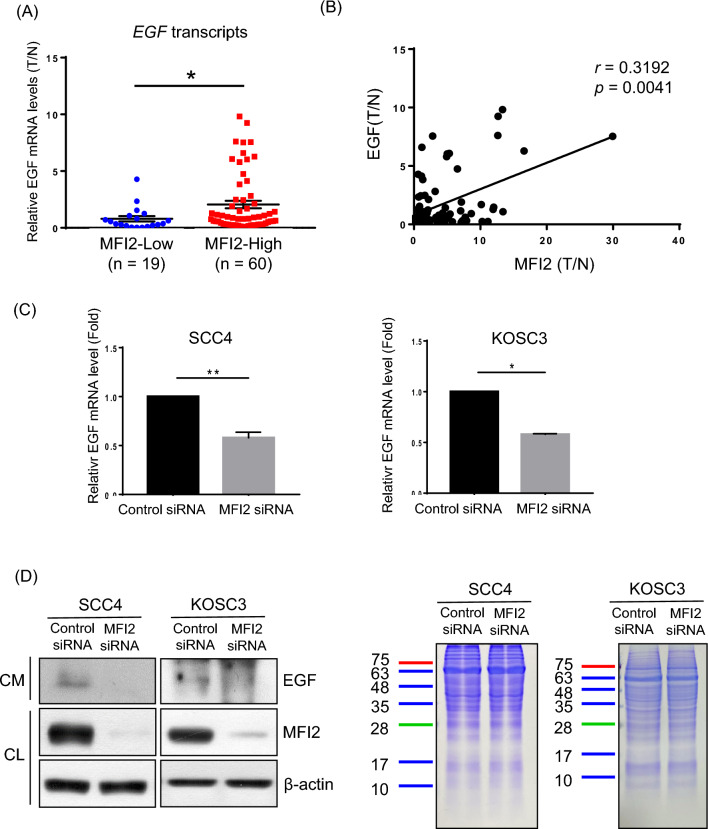
EGF positively correlates with MFI2 in OSCC. **A** Transcript expression levels of MFI2 from OSCC patient subgroups stratified by high and low MFI2 expression. **B** Correlation analysis between the transcript T/N fold of EGF and MFI2 from OSCC patients. **C** Gene expression of EGF in control and MFI2-specific siRNA-transfected SCC4 and KOSC3 cells was analyzed by qPCR. β-Actin was used as a normalized control. These data are representative of three independent experiments (*p < 0.05, **p < 0.01). **D** Western blot analyses of EGF and MFI2 in conditioned medium (CM) and cell lysate (CL), respectively (left). Representative SDS-PAGE stained by Coomassie blue showing total proteins in CM (right)

Growth factors regulate many signals for the homeostasis of tissues and their surroundings and act as major regulators of all subsequent steps of tumor progression [24, 25]. OSCC-associated growth factors were identified by multiplexed immunobead-based profiling in our previous study [26]. To explore the relationship between MFI2 and OSCC growth factors, the correlation between MFI2 and the growth factors was analyzed on cBioPortal based on the OSCC TCGA dataset. Epidermal growth factor (EGF), which is highly expressed in several types of cancer cells, including OSCC, was found to be among the top three genes positively correlated with MFI2 (Additional file 1: Figure S1), so the activation of EGF signaling might play a critical role in OSCC carcinogenesis. The mRNA levels of EGF from OSCC tumors were measured to determine the association between EGF and MFI2. As shown in Fig. 3A, the level of EGF mRNA was higher in the tumors with high expression of MFI2 than those with low expression of MFI2 (*p* = 0.0459). In addition, the level of MFI2 had a positive correlation with the level of EGF (*p* = 0.0041, *r* = 0.3192; Fig. 3B). These results implied that the expression of MFI2 influences the level of EGF. Additionally, we used another new cohort (total 107 samples) to identification. The mRNA levels of EGF were significantly higher in the OSCC patients with higher MFI2 expression and showed a significantly positive correlation between EGF and MFI2. This result has been added in Additional file 1: Figure S2. To prove this concept, we examined the mRNA level of EGF in MFI2-knockdown SCC4 and KOSC3 cells. As expected, the mRNA level of EGF was significantly decreased in MFI2-knockdown SCC4 and KOSC3 cells (Fig. 3C). As shown in Fig. 3D, the level of EGF in conditioned medium was markedly decreased from MFI2-knockdown SCC4 and KOSC3 cells compared with control cells. Collectively, these results suggest that knockdown of MFI2 reduced the level of EGF.

### Knockdown of MFI2 suppresses EGF-induced FAK phosphorylation in OSCC cells

**Fig. 4:**
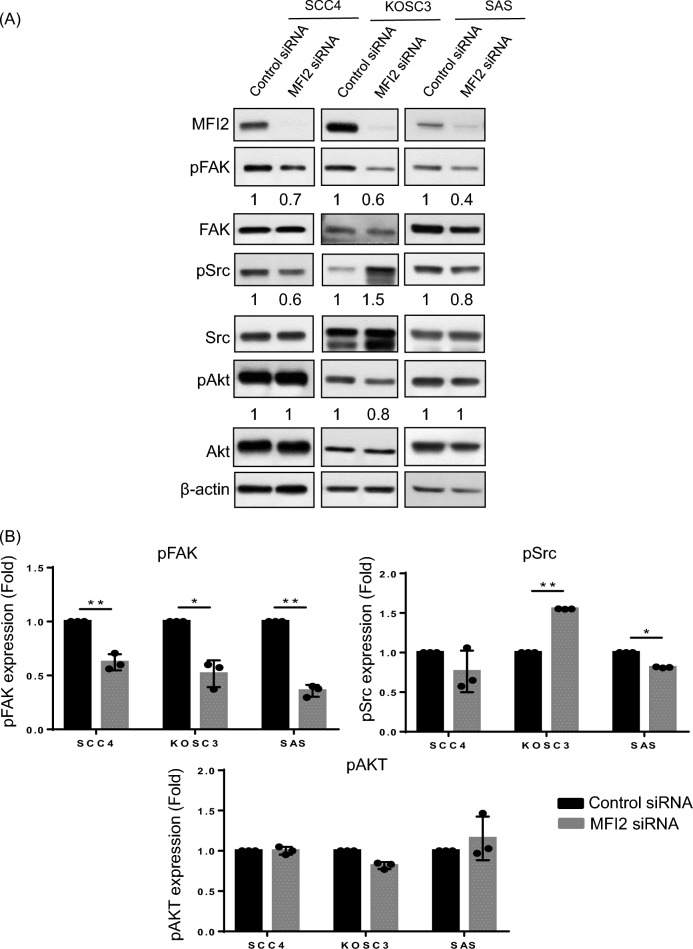

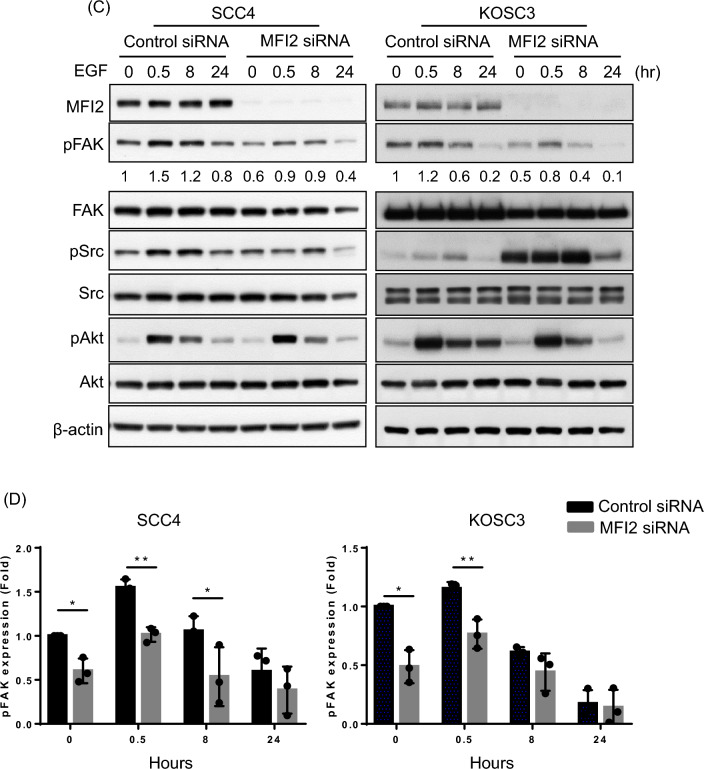
Phosphorylation of FAK is decreased in MFI2-knockdown SCC4, KOSC3 and SAS cells. **A** Control siRNA and MFI2-specific siRNA were transfected into SCC4, KOSC3 and SAS cells for 24 h and then incubated with serum-free medium for 24 h. Western blot analysis of the expression of MFI2, pFAK, FAK, pSrc, Src, pAkt, and Akt. β-Actin was used as a loading control. **B** Quantitative levels of pFAK, pSrc and pAkt in (**A**). The results are representative of three independent experiments (*p < 0.05, **p < 0.01). **C** Transfected cells were treated with EGF for 0.5, 8 or 24 h. Western blot of the expression of MFI2, pFAK, FAK, pSrc, Src, pAkt, and Akt. β-Actin was used as a loading control. **D** Quantitative level of pFAK in (**C**). The results are representative of three independent experiments (*p < 0.05, **p < 0.01)

EGFR is a tyrosine kinase, which leads to tyrosine autophosphorylation upon ligands binding. This triggers the phosphorylation of tyrosine kinases, followed by the initiation of a signaling pathway that activates various downstream signaling to mediate various cellular activities, including cell proliferation, cell survival, growth. Also, high expression of the EGF receptor is frequent event in human cancers that correlates with poor prognosis. EGF is a common mitogenic factor that elicits different downstream signaling pathways depends on different types of cancer or cell lines. PI3K/AKT signaling [27], focal adhesion kinase (FAK) [28] and Src/STAT pathway [29] are three of mainly EGF downstream pathways, which mediate cell motility and cell survival. According to Fig. 3, we found the expression level of EGF is decreased in MFI2-knockdown cells. To clarify which pathways are involved in MFI2-influened cancer progression, p-FAK, p-AKT and p-Src were determined in MFI2-knockdown OSCC cells. However, p-AKT had no significant different between control and MFI2 knockdown cells. Phosphorylation of Src have different changes in expression levels among three types of cells. This may be due to different types of cell lines have different effects. Among these downstream proteins, phospho-FAK was consistently downregulated in MFI2-knockdown SCC4, KOSC3 and SAS cells. (Fig. 4A and B).

To elucidate its specificity for the MFI2-mediated EGF/pFAK signaling pathway, additional EGF was administered at different time points, and the downstream molecules were observed. As expected, EGF treatment for 0.5 h induced the phosphorylation of FAK and Akt in both control and MFI2-knockdown SCC4 and KOSC3 cells. Notably, only phospho-FAK was decreased in MFI2-knockdown cells compared with control cells (Fig. 4C and D), suggesting that MFI2 is an important mediator for regulating the EGF-induced phosphorylation of FAK in OSCC cell lines.

### MFI2 promotes cell proliferation and migration through the EGF/pFAK signaling pathway

**Fig. 5:**
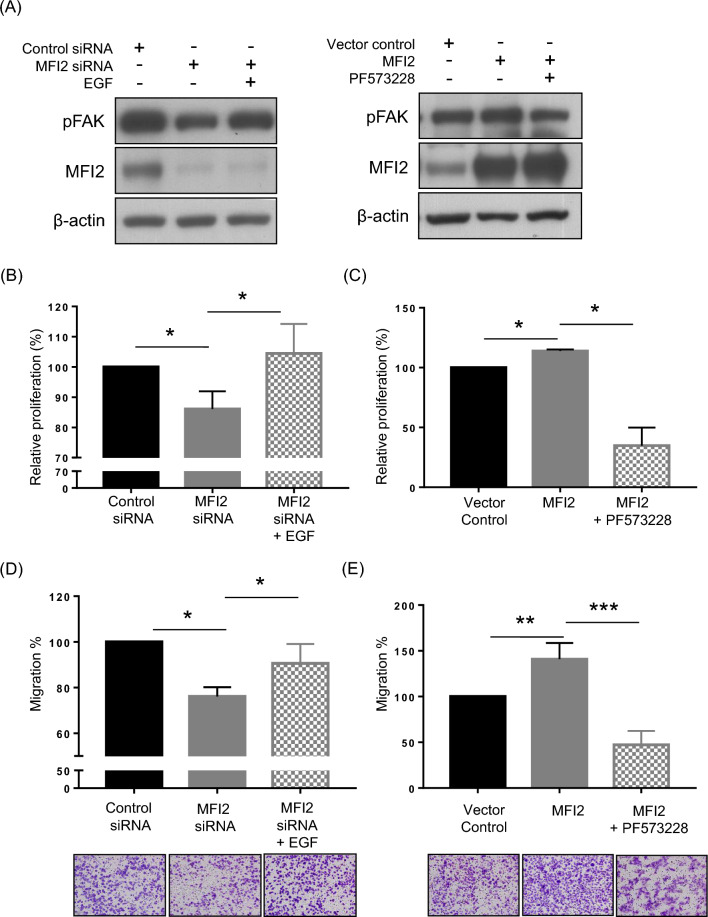
MFI2 promotes cell proliferation and migration through the EGF/pFAK signaling pathway. **A** Western blot of the MFI2 and pFAK expression in transfected KOSC3 cells. β-Actin was used as a loading control. MFI2-knockdown KOSC3 cells were treated with EGF (30 ng/ml). After transfection for 72 h, **B** cell proliferation and **D** migration were analyzed by CCK8 and Transwell assays, respectively. MFI2-overexpressing KOSC3 cells were treated with PF573228 (10 μM). After transfection for 72 h, **C** cell proliferation and **E** migration were analyzed by CCK-8 and Transwell assays, respectively. The results are representative of three independent experiments (*p < 0.05, **p < 0.01, ***p < 0.001)

The current study revealed that downregulated MFI2 reduced EGF/pFAK molecular signaling. We next investigated whether MFI2 influenced cell proliferation and migration through the EGF/pFAK signaling pathway. First, the transfection efficiency was confirmed, as shown in Fig. 5A. Next, a cell proliferation assay was performed in MFI2-knockdown and overexpression KOSC3 cells by CCK-8 assays. As shown in Fig. 5B, cell proliferation was reduced when MFI2 was knocked down, but this ability was rescued after EGF addition. Furthermore, cell proliferation was improved in MFI2-overexpressing KOSC3 cells, but this phenomenon was inhibited by pFAK inhibitor (PF573228) treatment (Fig. 5C). Transwell migration assays demonstrated that the invasion ability significantly decreased in MFI2-knockdown KOSC3 cells, but it was rescued after EGF treatment (Fig. 5D). Conversely, the invasion ability increased in MFI2-overexpressing KOSC3 cells, but it was inhibited by PF573228 treatment (Fig. 5E). Collectively, these data show that MFI2 overexpression predisposed OSCC patients to a worse prognosis by playing a vital role in the regulation of cell growth and motility through the EGF/pFAK signaling pathway.

## Discussion

In the current study, we identified dysregulated genes in OSCC by comparing the mRNA transcript abundance in the TCGA dataset with CNV in the OncoScan dataset according to our previous study [7]. Among 54 genes that were cross-matched in both datasets, 37 genes displayed a positive association with both CNV and mRNA transcript abundance. Genomic alterations drive carcinogenesis from genomic abnormalities to protein abundance and cancer phenotypes. Previously, Zhang et al. examined the impact of CNV on mRNA and protein abundance in colon and rectal cancer [30]. They calculated the correlation between all 23,125 genes and CNV in the TCGA dataset and mRNA and protein abundance. They found that the CNV-mRNA correlation revealed a strong effect and the CNV-protein correlation revealed a weaker effect [30]. Fan et al. also integrated CNV and differential gene expression by a bioinformatics approach across 1025 cell lines and 9159 patient samples [31]. They showed a close correlation between CNV and differential gene expression. These previous studies suggest that it is worth integrating multiomic data, such as genomic, epigenetic, and proteomic data, to help improve our current strategy of identifying dysregulated genes.

Recently, Lei et al. found that MFI2 consists of two forms: one is a membrane-bound protein (mMFI2) and the other is secreted out of the cell (sMFI2) in lung cancer, and the functions of the two forms are relatively independent [22, 32]. In addition, both mMFI2 and sMFI2 were associated with clinical outcomes, but the expression of sMFI2 was not significantly different between early stage cancer patients and normal volunteers. In the current study, MFI2 overexpression in OSCC tumor tissue or in OSCC cell lines (mMFI2) predisposed to a worse prognosis and played a vital role in modulating proliferation and mobility. This suggests that mMFI2 has an essential role in OSCC progression. To elucidate whether sMFI2 is also dysregulated in the body fluids of OSCC patients, we detected the level of sMFI2 in saliva from 100 healthy controls, 100 patients with oral premalignant disease and 200 OSCC patients by enzyme-linked immunosorbent assay. However, the level of MFI2 in saliva (sMFI2) was not significantly different among the three groups (Additional file 1: Figure S3). Accordingly, we speculated that mMFI2 may play a more dominant role than sMFI2 in OSCC tumorigenesis.

Herein, the current study revealed the correlation between MFI2 and EGF, and knockdown of MFI2 decreased the mRNA level of EGF in OSCC cell lines. It is interesting to investigate how MFI2 regulates the mRNA expression of EGF. To identify potential transcriptional regulatory factors for EGF, we used GeneHancer to define candidate enhancers of EGF [33] (http://www.genecards.org/) and identified 20 candidates. Based on the OSCC-TCGA database on the cBioPortal website (http://www.cbioportal.org/), we analyzed the correlation between the 20 candidates and MFI2. Five genes (DPF2, SOX12, ATF2, CREB3L4 and STAT5B) were positively correlated with MFI2, indicating that MFI2 may regulate the mRNA level of EGF by modulating these genes (data not shown). The detailed mechanism needs to be further investigated.

We found that silencing of MFI2 led to downregulation of FAK phosphorylation, which is one of the downstream of EGF. Interestingly, the silencing MFI2 does not significantly affect other EGF downstream targets, likes Akt and Src, indicating the modulating specificity in MFI2-regulating EGF/FAK signaling. Previous studies have indicated that several modulates can regulate specific EGF downstream targets, for example, TMEM16A, a dysregulated gene in many cancer types. TMEM16A can interact with EGFR and activate EGFR-signaling in HNSCC [34]. However, TMEM16A modifies the pattern of EGF-induced phosphorylation of EGFR without affecting Akt or Erk phosphorylation in pancreatic cancer [35]. Thus, the downstream effect appear to be molecular-dependence, underscoring the importance of investigating MFI2-dependent EGF/FAK-signaling in oral cancer. FAK is a ubiquitously expressed nonreceptor tyrosine kinase that significantly contributes to the upregulation of growth factor receptors, such as EGFR and PDGFR, and it integrates signals governing oncogenesis and tumor progression in cancer cells [36, 37]. It plays a significant role in cell survival, migration, invasion and metastasis of cancer cells, and the overexpression and activation of FAK have been reported in multiple types of human cancers, including HNSCC [38, 39]. Most importantly, Chiu et al. indicated that the immunoreactivity of FAK and FAK-pY397 was especially evident in metastatic oral lesions and positively correlated with the degree of malignancy by immunohistochemical staining, indicating that FAK and its phosphorylated form were associated with tumor invasion and metastasis in HNSCC [40]. In addition, Kato et al. showed that OSCC patients with high expression of FAK, FAK-pY397, or both had a significantly worse prognosis [41]. Several studies have also indicated the role of FAK in radiotherapy and chemotherapy resistance. Inhibition of the FAK-related pathway enhanced the chemosensitivity of OSCC, and overexpression of FAK was a biomarker for radioresistance in locally advanced HNSCC [42–44]. Collectively, these results suggest that FAK is involved in the tumorigenesis and progression of HNSCC and provides a therapeutic benefit to overcome tumor cell resistance to radiotherapy and chemotherapy.

## Conclusion

**Fig. 6:**
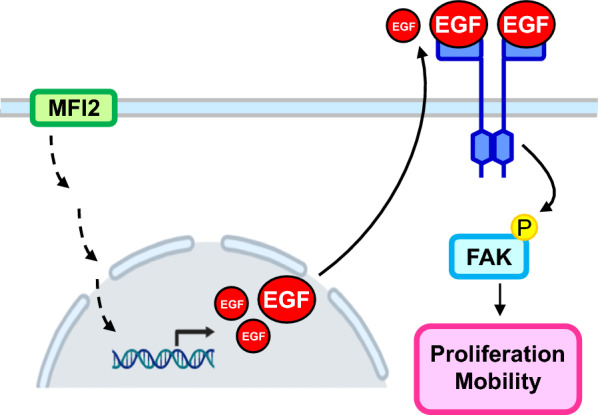
Hypothetical schematic of the role of MFI2 in OSCC malignant progression. In OSCC cells, an elevated MFI2 level triggers EGF-mediated pFAK signaling, which in turn increases cell proliferation and mobility, contributing to OSCC malignancy

Taken together, we intersected the genes with CNVs in the OSCC-OncoScan dataset that we previously developed and the DEGs from the OSCC-TCGA database to identify novel diagnosis- or prognosis-related genes in OSCC. MFI2 was selected as the target gene from among 11 candidates in this study through literature research and survival analysis. MFI2 overexpression led to a worse prognosis and played a vital role in modulating OSCC cell proliferation and mobility via the EGF/pFAK signaling pathway (Fig. 6).

## Supplementary Information

**Additional file 1**. Supplementary materials and figures.

## Data Availability

The data that support the findings of this study are available in the figures and the supplementary material of this article.
