# Supplementary material for: MFI2 upregulation promotes malignant progression through EGF/FAK signaling in oral cavity squamous cell carcinoma
Source: Cancer Cell Int. 2023 Jun 12;23:112. doi: 10.1186/s12935-023-02956-0 (PMC10259005; doi:10.1186/s12935-023-02956-0)
Supplement: Supplementary file 1 — Additional file 1. Supplementary materials and figures. [file 12935_2023_2956_MOESM1_ESM.docx]

**MFI2 upregulation promotes malignant progression through EGF/FAK signaling in oral cavity squamous cell carcinoma**

Wei-Chen Yen ^1, 2, #^, Kai-Ping Chang ^1, 2, 3, #^, Cheng-Yi Chen ^4^, Yenlin Huang ^5, 6^, Ting-Wen Chen ^7, 8, 9^, Hsing-Wen Cheng ^1^, Jui-Shan Yi ^1^, Chun-Chia Cheng ^10^, Chih-Ching Wu ^1,2,11^, Chun-I Wang ^2, *^

**Additional materials**

**Enzyme-linked immunosorbent assay for MFI2 determination in OSCC saliva**

The MFI2 levels in the saliva of 100 healthy controls, 100 oral potentially malignant disorders (OPMD) and 200 OSCC patients were measured using a commercially available ELISA kit (Cloud Clone Corp., Houston, TX, USA). ELISAs were performed according to the manufacturer’s instructions. Briefly, 100 μl of saliva sample was added to each well and incubated for 2 hours at 37 °C. Then, 100 μl of reagent A was added for an additional hour. After 3 washes, reagent B was added for 30 minutes. The amount of protein was determined by adding 3,30,5,50-tetramethylbenzidine (TMB) substrate, and the plate was incubated at 37 °C for color development. The absorbance was measured at 450 nm using a SpectraMax M5 microplate reader (Molecular Devices, Sunnyvale, CA).

**Additional figures**


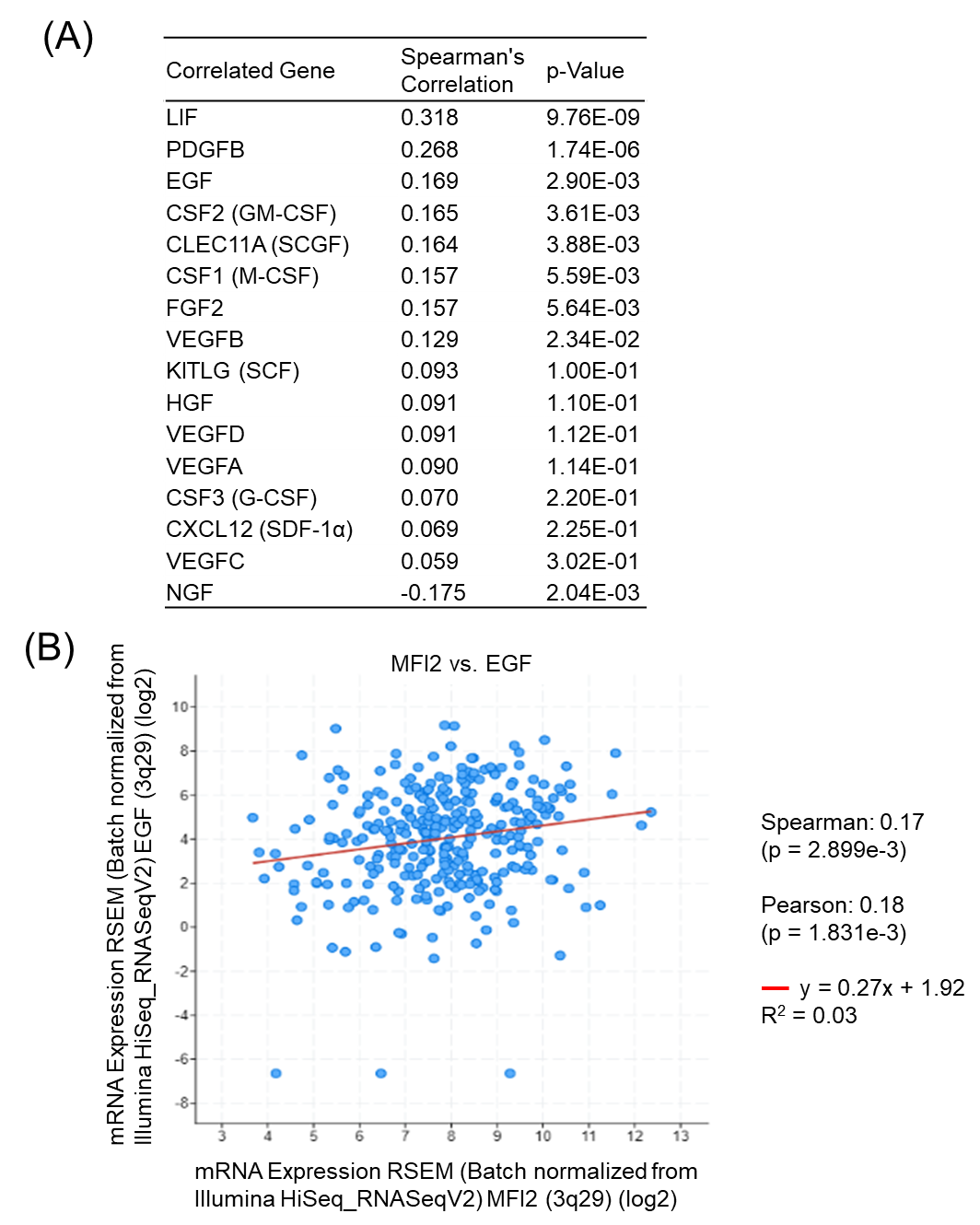


**Figure S1. The correlation analysis of growth factors and MFI2.** (A) The correlation between MFI2 and OSCC associated-growth factors was analyzed on cBioPortal based on OSCC-TCGA data set. (B) The correlation analysis of EGF and MFI2 on OSCC-TCGA data set.


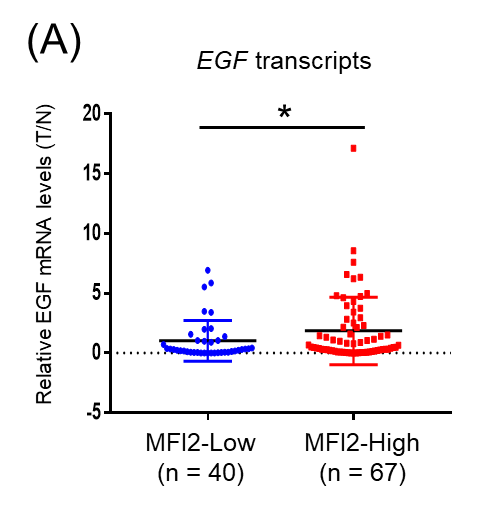


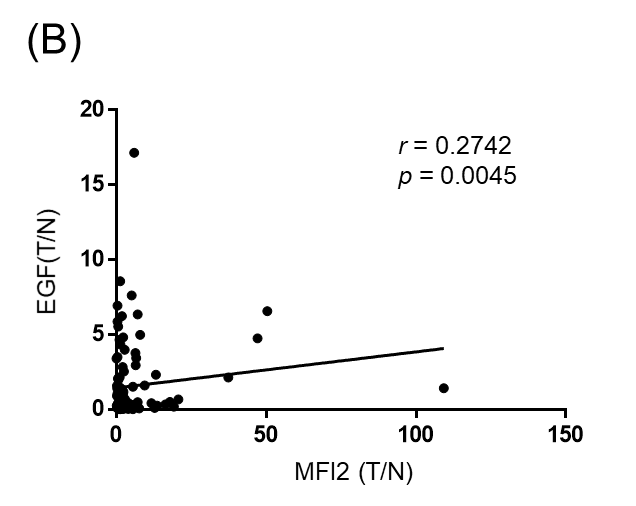


**Figure S2. EGF positively correlates with MFI2 in OSCC.** (A) Transcript expression levels of MFI2 from OSCC patient subgroups stratified by high and low MFI2 expression. (B) Correlation analysis between the transcript T/N fold of EGF and MFI2 from OSCC patients.


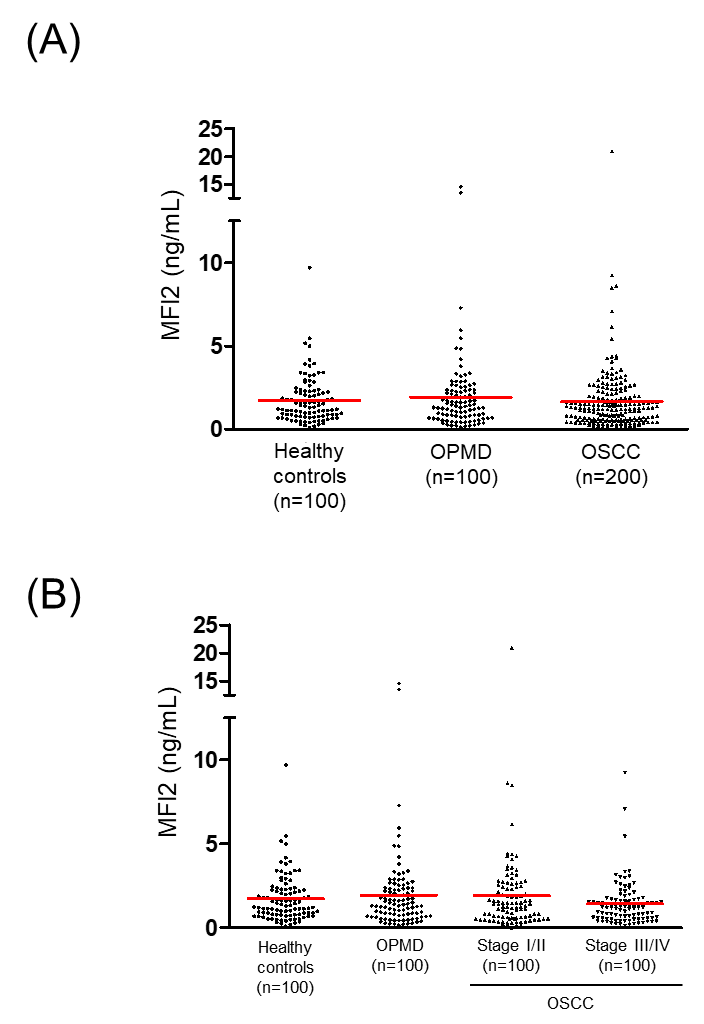


**Figure S3. The level of MFI2 from saliva of individuals.** (A) Comparison the level of MFI2 from saliva among healthy, OPMD and OSCC patients. (B) Comparison the level of MFI2 from saliva among healthy, OPMD and early- and late-stage of OSCC patients. The level of MFI2 was determined by ELISA kit.
